# Introduction of a three-dimensional computed tomography measurement method for acetabular fractures

**DOI:** 10.1371/journal.pone.0218612

**Published:** 2019-06-19

**Authors:** A. M. L. Meesters, J. Kraeima, H. Banierink, C. H. Slump, J. P. P. M. de Vries, K. ten Duis, M. J. H. Witjes, F. F. A. IJpma

**Affiliations:** 1 Department of Surgery, Subdivision of Trauma Surgery, University Medical Center Groningen, University of Groningen, Groningen, The Netherlands; 2 3D lab / Department of Oral and Maxillofacial Surgery, University Medical Center Groningen, Groningen, The Netherlands; 3 MIRA Institute for Biomedical Engineering and Technical Medicine, University of Twente, Enschede, The Netherlands; 4 Department of Surgery, University Medical Center Groningen, University of Groningen, Groningen, The Netherlands; Kanazawa University, JAPAN

## Abstract

**Introduction:**

Acetabular fractures consist of complex fracture patterns whereby bone fragments are displaced in different directions. Two-dimensional computed tomography (2DCT) gap and step-off measurements tend to underestimate the multidirectional features of these fractures. The aim was to develop a three-dimensional computed tomography (3DCT) measurement method for acetabular fractures and validate whether this method will provide an observer independent fracture characterization.

**Materials and methods:**

Sixty patients, operated for an acetabular fracture between 2007 and 2018, were included. The displacement was measured on the pre- and postoperative CT scans. Pre- and postoperative CT-based 3D models were made for each patient. Multiple 3D measurements, namely the 3D step-off, gap and the total gap area were introduced to quantify the preoperative and postoperative displacement. The Wilcoxon signed rank analysis was used to compare the 2DCT and 3DCT measurements.

**Results:**

The preoperative displacement was significantly underestimated by 2DCT measurements in comparison with 3DCT measurements (2D vs. 3D; step-off 8 vs. 16 mm with P < 0.001; gap 19 vs. 21 mm with P = 0.001). The same applies to the postoperative residual displacement (2D vs. 3D; step-off 0 vs. 6 mm; gap 3 vs. 8 mm; P < 0.001). The total gap area, defined as the surface area between all fracture lines in the 3D model, was measured for each patient, resulting in a median value of 722 mm^2^ preoperatively and 168 mm^2^ postoperatively, with excellent inter- and intra-rater reliability.

**Conclusion:**

2DCT measurements tend to underestimate the initial and residual displacement in complex acetabular fractures. A 3DCT analysis of these injuries was developed to overcome this and should be used in addition to the Judet/Letournel and AO/OTA classification systems, in order to provide an observer independent quantifiable fracture description and accurate assessment of the fracture reduction.

## Introduction

Acetabular fractures are frequently composed of complex fracture patterns whereby multiple bone fragments are displaced in different directions. Understanding these aspects of displacement is mandatory for a surgeon to determine the optimal treatment strategy. [1] The Judet and Letournel as well as the AO/OTA are the most widely used classification systems for acetabular fractures. [2–4] Moreover, Matta’s grading system is principally used to determine the residual postoperative displacement. [5]

It is generally not possible with the current imaging modalities, namely plain radiographs and/or subsequent CT slices, to get a complete picture of the three-dimensional aspects of the fracture. The current classification systems represent the gross fracture patterns, but they do not include quantitative information about the displacement of each fracture fragment. Despite the availability of advanced three-dimensional imaging techniques, the displacement of fracture fragments is still often determined on plain radiographs or 2D projections of CT slices. The physician’s assessment of the CT scan is influenced by how the gap and step-off measurements are performed and which CT slice is selected for these measurements. Therefore, these 2D measurements of the axial, coronal and sagittal CT slices might vary substantially between different observers. Moreover, it is challenging to objectify the true extent of the injury from 2DCT slices, since the articular incongruity frequently involves displacement of fracture fragments in multiple planes, along multiple fracture lines in a concave anatomical structure. 3D technology, however, has the potential advantage to represent this multidirectional feature of acetabular fractures. [6–8]

Unfortunately, no uniform 3D measurement technique is available to determine the degree of initial fracture displacement and the subsequent amount of postoperative reduction in acetabular fracture treatment. The aim of this study, therefore, is to develop and validate a 3DCT measurement method for the analysis of acetabular fractures. We hypothesize that quantifying acetabular fractures, by means of pre- and postoperative 3D measurements, will provide an observer independent fracture characterization and an accurate postoperative assessment of fracture reduction.

## Materials and methods

### Patients

Patients, who were treated with open reduction and internal fixation for a unilateral acetabular fracture at a level 1 trauma center, between November 2007 and May 2018, were eligible for inclusion in this study. Sixty randomly selected patients were included in this study, based on the availability of a complete dataset of high quality pre- and postoperative CT scan (with a maximum slice thickness of 2 mm). Patient demographics were retrieved from the patient files (Table 1). Two trauma surgeons collectively graded all the fractures according to the AO/OTA and Judet and Letournel classification systems by reassessing all the preoperative radiographs and CT scans. [4] Additionally, the maximum fracture step-offs and gaps in all patients’ acetabular domes were measured on axial, coronal and sagittal CT slices, in consensus by 3 observers (2 trauma surgeons and 1 technical physician), according the method as described by Verbeek et al. method 2018. [9] This study was reviewed and a waiver was provided by the local Medical Ethics Review Committee, no: 2016.385.

**Table 1:** Patient characteristics.

Patient demographics (N = 60)	
**Sex (no.)**	
Male	51
Female	9
**Mean age (range) (yrs)**	49 (19–81)
**Trauma mechanism (no.)**	
High energy trauma	34
Low energy trauma	26
**Classification AO/OTA (no.)**	
A	20
B	19
C	21
**Classification Letournel (no.)**	
Elementary fracture types	16
Posterior wall	12
Anterior column	3
Transverse	1
Associated fracture types	44
Posterior column and wall	4
Transverse and posterior wall	9
T-shaped	5
Anterior column/wall with posterior hemitransverse	5
Both columns	21

### 3D models

**Fig 1:**
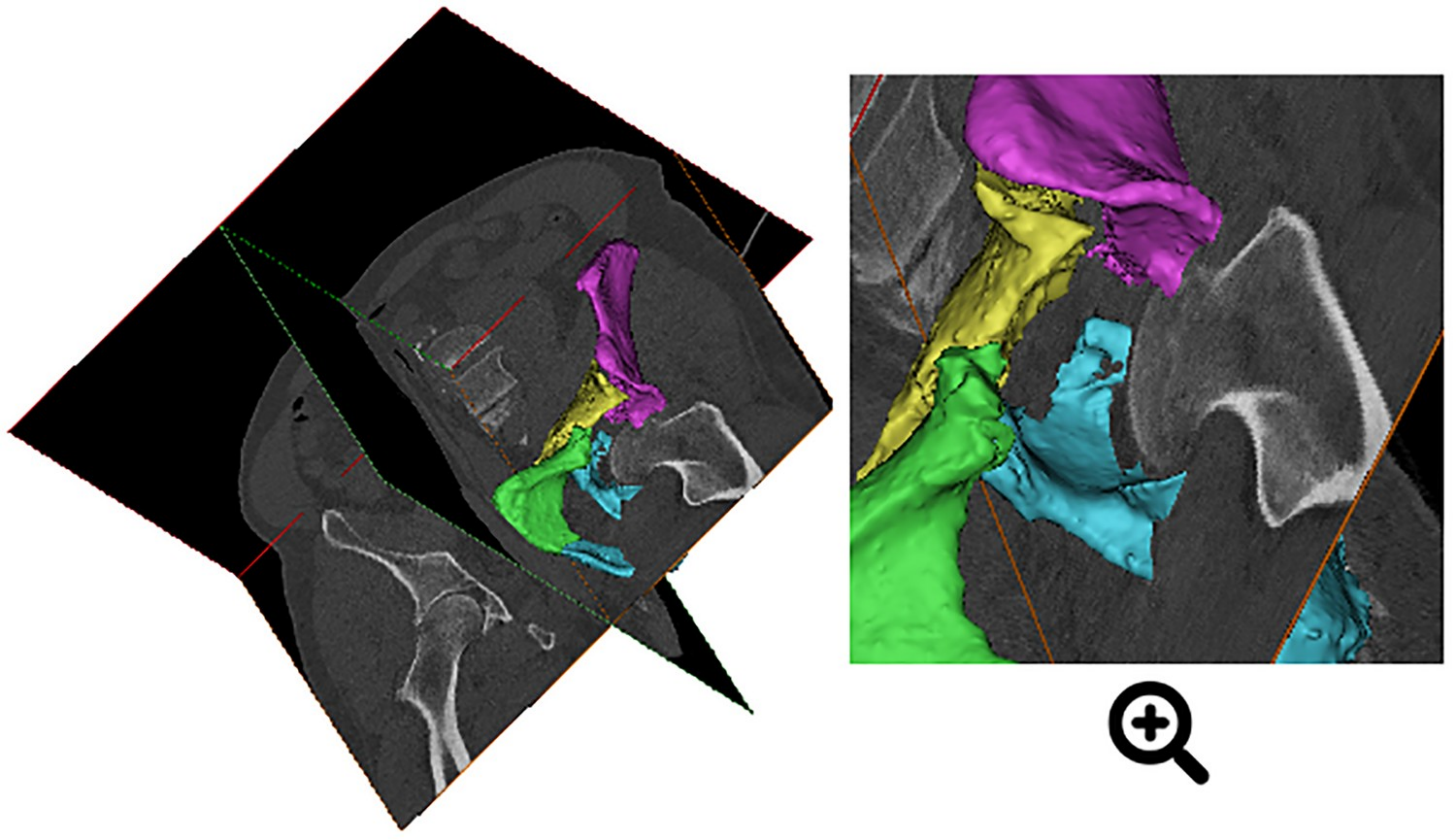
Visualization. Dual projection of a preoperative CT scan of an acetabular fracture with a superimposed 3D model, generated from the CT data.

The CT data (maximum slice thickness of 2 mm) was imported into the Mimics Medical software (version 19.0; Materialise, Leuven, Belgium). A 3D model of the pelvis was generated by segmentation of the bone tissue (Fig 1), using a preset threshold for bone. The unilateral fracture fragments were identified. Subsequently, virtual anatomical reduction of the fragments was performed by using the mirrored intact acetabulum, of that same patient, as a template (3-matic Medical software, version 11.0; Materialise, Leuven, Belgium). The preoperative fragments were matched with the postoperative 3D model, to determine the postoperative fragment locations within the acetabulum. An animation, in which the 3D models and measurements methods are clarified, is included as: S1 Video.

### Quantitative 3DCT measurements

All measurements were performed on the pre- as well as the postoperative 3D model, using 3-matic Medical software (version 11.0; Materialise, Leuven, Belgium). First, the conventional step-offs and gaps were measured in 3D. Furthermore, an additional parameter, the total gap area was introduced. The method of measuring of each parameter is described in the following sections.

#### 3D step-off and gap

**Fig 2:**
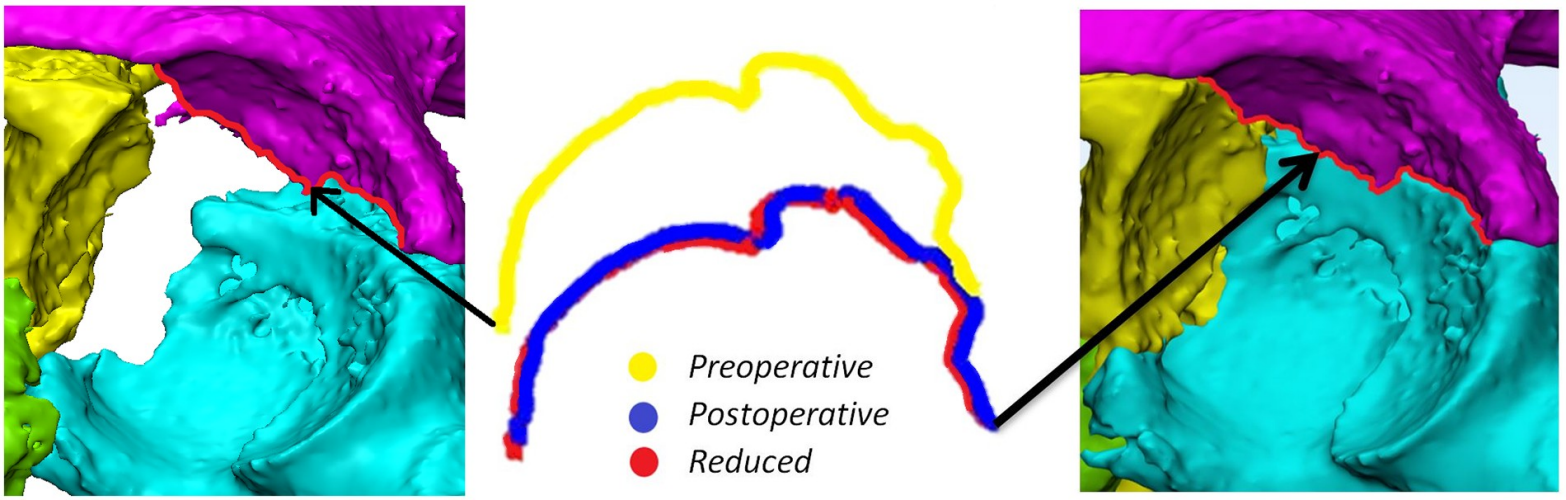
Measurement of the 3D step-off and 3D gap along one of the fracture lines. Left: Fracture lines on the preoperative 3D model. Middle: Representation of the preoperative (yellow), postoperative (blue) and the virtual reduced (red) fracture lines. Right: Location of the fracture lines on the postoperative 3D model.

The 3D step-offs and gaps were determined by measuring the inter-fragment distances perpendicular to and along the articular surface. Fracture lines were drawn along the edges of each fragment in the preoperative 3D fracture model as well as the postoperative model (Fig 2). The 3D displacement was defined as the amount of displacement along the x-, y- and z-axis for each fragment in a three-dimensional space. The reduced 3D fracture model was used as a reference. The 3D distance between the pre-/postoperative and the reduced fracture lines was calculated (in Matlab R2016B, Mathworks Massachusetts, US), for every point on the fracture line, according to the Euclidean distance formula. The 3D step-off was defined as the (3D) displacement of the fracture lines perpendicular to the articular surface. The 3D gap was defined as the (3D) displacement of the fracture lines along the articular surface. The maximum and average values of the step-offs and gaps were calculated.

#### Total gap area

**Fig 3:**
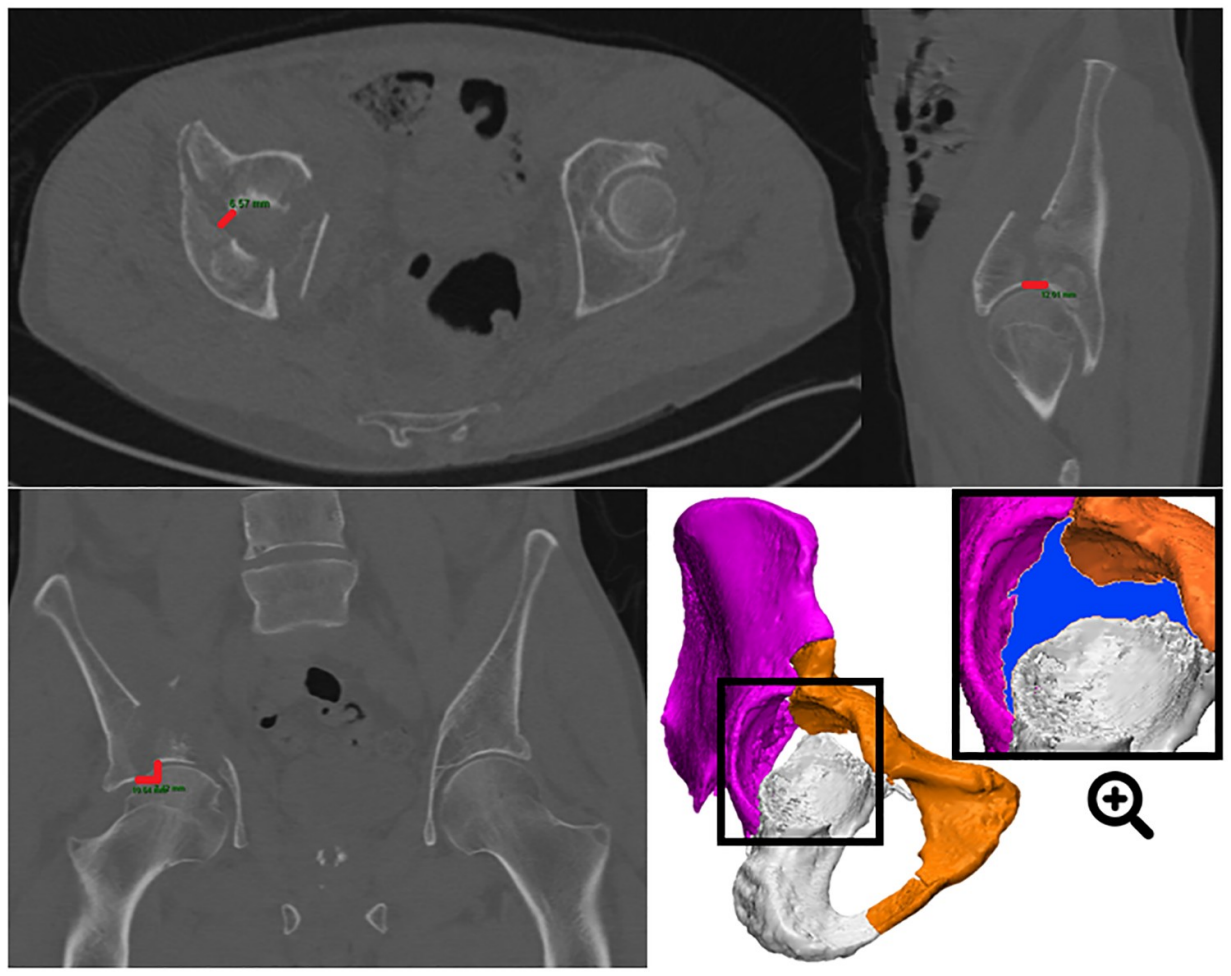
Case example showing both 2DCT and 3DCT measurements of an anterior column and posterior hemitransverse type acetabular fracture with multiple fracture lines going in different directions. 2DCT measurements were performed on the preoperative axial (step-off 0 mm; gap 7 mm), coronal (step-off 7 mm; gap 11 mm) and sagittal (step-off 0 mm; gap 12 mm) CT slices, resulting in a maximum 2D step-off of 7 mm and gap of 12 mm. The direction of measuring the maximum gap is arbitrary, particularly on the axial CT slice. 3DCT measurements, performed on a preoperative 3D model, resulted in a larger maximum gap (2D vs. 3D; 12 vs. 16) and step-off (2D vs. 3D; 7 vs. 16). The total gap area, defined as the surface area between all fracture lines, is marked in blue on the preoperative 3D model.

The total gap area is defined as the surface area between all fracture lines at the articular surface (Fig 3). It was measured when analyzing the acetabulum from a standardized point of view. A plane was created, on the mirrored healthy acetabulum, using the anterior inferior iliac spine, the lowest and most lateral point of the foramen and the most prominent point of the ramus superior, as landmarks. This plane allowed a standardized orientation of the acetabulum, perpendicular to the plane. In this visualization, the preoperative and postoperative total gap areas were measured in mm^2^.

### Statistical analysis and validation

The Wilcoxon signed rank test was used to determine statistical differences between the maximum 2D and 3D measurements. The Spearman’s correlation coefficient, ρ (rho), was used to determine the strength of association between the 2D and 3D measurements. A p-value lower than 0.05 was considered significant.

Certified software, making one-on-one translations from the CT to the 3D model, was used to guarantee the accuracy of the 3D measurements. Additionally, a validation test was performed, using a printed pelvis (S1 Text). Furthermore, the pre- and postoperative total gap areas, of 10 randomly selected patients, were measured by two independent observers (technical physicians) to test the inter-observer variability. Additionally, one observer repeated the measurements on these ten patients’ models to calculate the intra-observer variability. The reliability was assessed in SPSS (version 23, IBM, Chicago, IL, US), using the Intraclass Correlation Coefficient (ICC), with a two-way mixed, single measurements model with absolute agreement.

The residual displacement of fragments in the postoperative 3D model was graded according to Matta’s criteria. [5] Additionally, the percentage of reduction was calculated for the average 3D step-off, average 3D gap and the total gap area by calculating the difference between the pre- and postoperative measurements. The overall postoperative reduction was calculated by averaging these percentages of reduction.

## Results

### Validation test

The validation test showed that the 2D gap and step-off underestimate the actual gap and step-off with 2–5 mm (S1 Text). Additionally, the 3D gap and step-off measurements had a deviation of less than 1 mm with the actual values.

### Step-offs and gaps in 2D and 3D

The median preoperative step-off was 8 in 2D vs. 16 mm in 3D and the median preoperative gap was 19 vs. 21 mm. Therefore, the amount of preoperative displacement (Table 2) was significantly underestimated (P < 0.001 step-off; P = 0.001 gap) by the 2D measurements compared to the 3D measurements. Postoperatively, the median step-off was 0 vs. 6 mm and the gap was 3 vs. 8 mm in 2D vs. 3D. Thus, the degree of postoperative residual displacement was significantly underestimated (P < 0.001) by the 2D measurements in comparison with the 3D measurements. Additionally, there was a moderate correlation between the 2D and 3D measurements (Table 2). Traditionally, the degree of displacement is determined by the greatest gap or step-off in any of the axial, sagittal, or coronal CT slices. [9] Instead of taking the highest value in one of the 2DCT slices, 3D measurements have an additional feature, namely to measure the average 3D step-off and gap along all fracture lines, which is a more extensive representation of the fractured acetabulum as a whole. 2D measurements slightly underestimate the amount of residual displacement in comparison to the average 3D measurements (step-off 0 vs. 2 mm; gap 3 vs. 4 mm) (Table 3). The results of the measurements for all individual patients are added as S1 Table.

**Table 2:** Pre- and postoperative displacement.

Measurements		2D^a^	3D^a^	p-value^b^	Correlation^c^
**Preoperative** **initial displacement**	Gap (mm)	19 [12–27]	21 [16–36]	P < 0.001	0.4
	Step-off (mm)	8 [4–15]	16 [11–24]	P < 0.001	0.5
**Postoperative** **residual displacement**	Gap (mm)	3 [2–5]	8 [5–11]	P < 0.001	0.3
	Step-off (mm)	0 [0–2]	6 [4–8]	P = 0.001	0.5

Determining the amount of preoperative initial displacement and postoperative residual displacement, as measured by the maximum gap and step-off in 2D and 3D.

^a^: Median [inter quartile range].

^b^: Differences in the 2D and 3D measurements were tested with the Wilcoxon signed rank test.

^c^: The Spearman’s correlation coefficient was used to test the degree of association between the 2D and 3D measurements.

**Table 3:** Quantification of postoperative reduction through 2D and 3D measurements.

Measurements		Preoperative^a^	Postoperative^a^	% Residual displacement^b^
**2D**	Gap (mm)	19 [12–27]	3 [2–5]	19%
	Step-off (mm)	8 [4–15]	0 [0–2]	0%
	**Overall reduction**^c^	n/a	n/a	15%
**3D**	Gap (mm)	12 [9–16]	4 [3–6]	36%
	Step-off (mm)	7 [4–8]	2 [2–3]	39%
	Total gap area (mm^2^)	722 [451–1030]	168 [60–283]	22%
	**Overall reduction**^c^	n/a	n/a	35%

^a^: Median [inter quartile range]. 2D measurements are represented by the maximum step-off and gap. 3D measurements include the average 3D step-off, 3D gap and total gap area.

^b^: Median percentages. A percentage of 0 indicates anatomical reduction.

^c^: The 2D overall reduction was calculated by averaging the percentages of the step-off and gap reductions for every patient. The 3D overall reduction was calculated by averaging the percentages of the 3D step-off, 3D gap and total gap area reductions for all patients.

### Additional 3D measurements

The preoperative total gap area, defined as the surface area between all fracture lines in the 3D model, was measured for each patient and resulted in a median gap area of 722 mm^2^ (Table 3). After reducing the fracture fragments during surgery, the total gap area was determined again for each patient; it demonstrated a substantially lower median total gap area of 168 mm^2^. In daily practice, the postoperative residual fracture gap is mostly determined on a single CT slice (Fig 3). Instead, the total gap area represents the gap of the entire fracture and can be used as a standardized quantitative measure for the postoperative reduction. The inter-rater (ICC 0.99 and an absolute mean difference of 39.6 mm^2^ preoperatively; ICC 0.97 and an absolute mean difference of 39.9 mm^2^ postoperatively) as well as the intra-rater reliabilities (ICC 0.99 and an absolute mean difference of 28.5 mm^2^ preoperatively and 24.4 mm^2^ postoperatively) of the total gap area were excellent.

### Postoperative reduction according to Matta’s criteria

**Fig 4:**
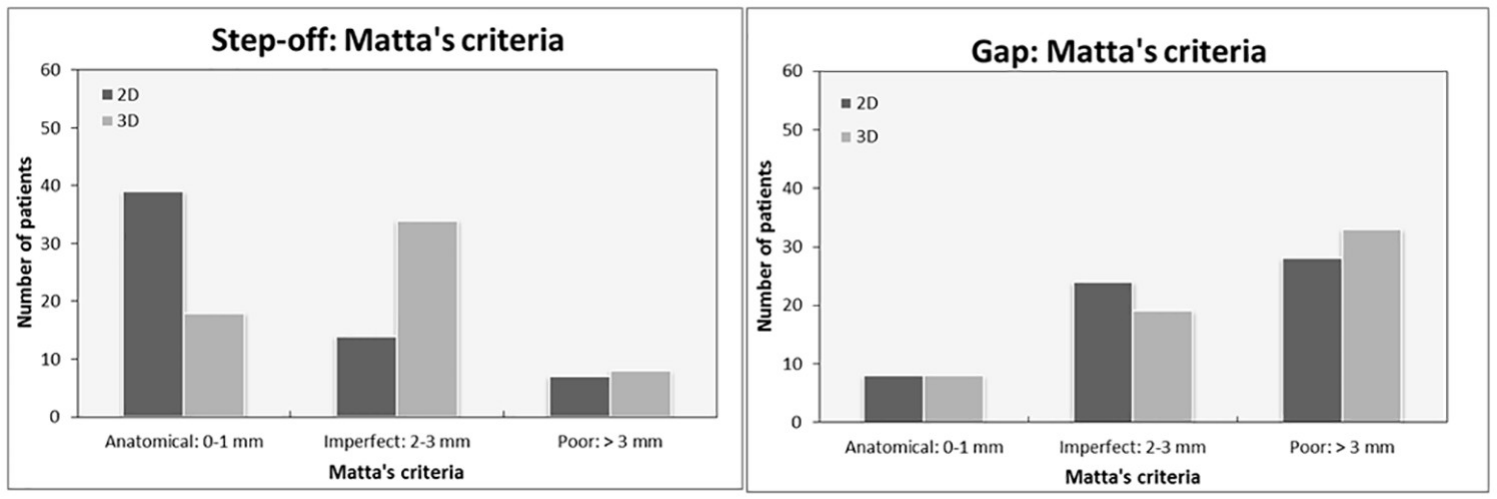
Quantification of the postoperative reduction in 2D and in 3D. The residual step-off (A) and the residual gap (B), graded according to Matta’s criteria.

The residual step-off was underestimated in the 2DCT in comparison with the 3D measurements (Fig 4). The 2DCT measurements graded the majority of patients (N = 39) as having an anatomical reduction whereas the 3D measurements graded most of them (N = 34) as having imperfect reductions (Fig 4A). The residual gaps of the 2D as well as the 3D measurements exceed Matta’s x-ray criteria, which would have graded the majority of patients as having poor reductions (Fig 4B).

A complete protocol for the 3D analysis, with all the measurements of a patient treated for an acetabulum fracture, can be found in: S2 Text.

## Discussion

A quantitative 3DCT measurement method was developed for the analysis of acetabular fractures, because 2DCT measurements underestimate the amount of initial and residual displacement. This study demonstrates that accurate and reliable intra-articular gap and step-off measurements in 3D are feasible in acetabular fracture surgery. Moreover, the total gap area is presented, in order to quantify the amount of displacement in a multitude of fracture lines. These 3D measurements should be used in addition to the Judet/Letournel and AO/OTA in order to quantify the amount of pre- and postoperative fracture displacement.

However, the correlation between the 2DCT and 3DCT measurements was moderate. An explanation for this is that the 2DCT measurements outcomes rely heavily on the physician’s subjective slice selection. The maximum gap and step-off are arbitrary representatives of the complete injury. It is often challenging to determine which gaps and step-offs are the largest in case of multiple fracture lines (Fig 3). Also, CT slices might not be orientated perpendicularly to the fracture line and therefore misjudge the 2DCT gap or step-off measurements. 3DCT measurements do not rely on challenges with slice selection, slice orientation, limited measurement points and selection bias in case of multiple fracture lines. Furthermore, the 3DCT method has the ability to not only calculate the maximum step-offs and gaps but also the average steps-offs and gaps along all the fracture lines. The inter- and intra-rater variability between 3D measurements was low in the current analysis.

As an additional 3D parameter, the total gap area was introduced. The gap area of the complete fracture, which included all fracture lines (Fig 3), was found to be observer independent and reliable (ICC 0.99). It comprises the gap of the complete fracture, including all fracture lines (Fig 3). It has some potential advantages over the 2DCT single slice maximum gap. Discrepancy between the gap area and the conventional gap can be attributed to different sized gaps in multiple fracture lines. For instance, the 3DCT total gap area might be quite large compared to the 2DCT gap in the case of multiple fracture lines with moderate gapping (S1 Table, patients 2, 35, 38, 45, 47 and 60).

The quality of the reduction is currently mostly graded according to the Matta criteria. [5] This grading system was developed by Matta in the 90s whereby the largest residual gap or step-off on plain postoperative radiographs in 3 views was used to determine the quality of the reduction (anatomical reduction, 0–1 mm; imperfect, 2–3 mm; or poor, > 3 mm). The majority of patients had a residual gap of > 3 mm after surgery and, according to Matta’s criteria, should have been graded as poor reductions (Fig 4). However, it should be noted that the postoperative CT scanning technique used in the current study is more accurate for detecting residual displacement than conventional x-rays on which the grading system and the valuation of the postoperative reduction is based. These finding are consistent with the Verbeek et al. results. [9,10] A uniform CT-based system for grading the amount of postoperative residual displacement is lacking. Therefore, a CT-based system for Matta criteria is needed. It is interesting to hypothesize what new 3DCT Matta-type criteria for defining residual displacement should consist of. First of all, a postoperative CT scan usually demonstrates increased amount of residual displacement after surgery compared to a postoperative pelvic radiograph. [11] Second, we are concerned whether a single gap or step-off measurement on a 2DCT slice should be considered a good representative of the entire fractured acetabulum that usually consists of multiple fracture lines and displacements in different directions. Third, the original criteria do not make a distinction between gap and step-off displacement. Verbeek et al. recently reported that residual gaps, as assessed on CT imaging, are better tolerated than step-offs in terms of long-term functional outcome of the hip joint. [10] Our preliminary 3DCT results (S1 Table) can be divided into 3 categories representing a ‘perfect, good or moderate’ reconstruction of the fractured acetabulum. Furthermore, the increased amount of residual displacement on CT measures compared to radiographs, the distinction between gaps and step-offs and the additional value of the (3D) total gap area were taken into account. Therefore, the following ***3DCT reduction criteria*** could be suggested: 1. For the gap, perfect reduction in case of 0–2 mm of residual displacement, good 2–5 mm and moderate > 5 mm; 2. For the step-off, perfect reduction in case of 0–1 mm of residual displacement, good 1–3 mm and moderate >3 mm; 3. For the total gap area, perfect reduction in case of 0–100 mm^2^ of residual displacement, good 100–200 mm^2^ and moderate > 200 mm^2^. The average grade of all three parameters can be considered the final assessment of the quality of the reduction.

### Strengths and limitations

This study presents an innovative 3D analysis that uses the traditional 2D measurements as a reference. The new method provides a 3D assessment of the whole acetabulum, including all individual fracture lines. The 3DCT method has also got certain limitations. Performing a 3D acetabular fracture analysis requires 3D software and the expertise of technical physicians and engineers, which are not available in all hospitals. That is why the next step after validation is optimization of the workflow, using automatic bone segmentation and automatic analysis of the fracture, which makes it easier to implement the 3D acetabular fracture analysis in other hospitals.

Controversy exists about routine use of postoperative CT scans, because of the costs, radiation exposure and limited clinical consequences. [12,13] Nevertheless, routine postoperative CT scans have been used in large studies for assessing postoperative reduction, determining patient’s prognosis, and the evaluation of the surgical techniques. [9] There is no difference in radiation exposure between 3DCT and 2DCT fracture assessment, because both measurement techniques are based on the same CT data. Furthermore, the use of postoperative CT scanning will gradually shift towards intraoperative CT scanning to optimize surgical outcome and therefore this 3D acetabular fracture analysis can also be used for intraoperative CT in the near future.

### Implications for current practice

Based on the study findings we recommend using the 3DCT method to measure the pre- and postoperative displacement in surgically treated acetabular fractures, instead of only using CT slices. The purpose of this study was to present and validate the new measurement method and not to correlate the results with the clinical outcome. Clinical studies in which the 3DCT measurements will be associated with patient reported outcomes are being conducted.

In conclusion, we present a validated quantitative 3DCT analysis of acetabular fractures, which is reliable, observer independent and should be used in addition to the current classification systems to assess preoperative initial displacement and the quality of the postoperative reduction.

## Supporting information

S1 Video Clinical case example of a 3D pelvis. Video that clarifies the 3D models and measurements methods.
(MP4)

S1 Table Measurements. Tables with the results of the measurements for all individual patients.
(DOCX)

S1 Text Validation. Validation of the gap and step-off measurements.
(DOCX)

S2 Text Protocol. A complete protocol for the 3D analysis, with all the measurements of a patient treated for an acetabulum fracture.
(DOCX)
