# Supplementary material for: Introduction of a three-dimensional computed tomography measurement method for acetabular fractures
Source: PLoS One. 2019 Jun 19;14(6):e0218612. doi: 10.1371/journal.pone.0218612 (PMC6583999; doi:10.1371/journal.pone.0218612)
Supplement: S1 Table — Tables with the results of the measurements for all individual patients. (DOCX) [file pone.0218612.s002.docx]

# Supplementary materials II: Individual results

The 2D step-offs and gaps of all the patients are shown in Table A1. The results of the 3D measurements (the 3D step-off, 3D gap and the total gap area) and the 3D overall reduction are shown in Table A2.

Table A1: The pre- and postoperative 2D step-offs and gaps of 60 patients.

|  | **2D step-off** | | | **2D gap** | | | **Overall** |
| --- | --- | --- | --- | --- | --- | --- | --- |
| **Patient** | **Pre-operative (in mm)** | **Post-operative (in mm)** | **Reduction (in %)** | **Pre-operative (in mm)** | **Post-operative (in mm)** | **Reduction (in %)** | **Reduction (in %) *** |
| 1 | 13 | 0 | 100 | 45 | 3 | 94 | 97 |
| 2 | 4 | 2 | 50 | 21 | 10 | 52 | 51 |
| 3 | 21 | 0 | 100 | 34 | 14 | 59 | 79 |
| 4 | 19 | 1 | 95 | 28 | 5 | 82 | 88 |
| 5 | 4 | 2 | 50 | 19 | 5 | 74 | 62 |
| 6 | 3 | 1 | 67 | 19 | 3 | 84 | 75 |
| 7 | 12 | 0 | 100 | 22 | 3 | 86 | 93 |
| 8 | 21 | 2 | 90 | 26 | 4 | 85 | 88 |
| 9 | 9 | 12 | -33 | 42 | 15 | 64 | 15 |
| 10 | 10 | 0 | 100 | 20 | 3 | 85 | 93 |
| 11 | 11 | 0 | 100 | 28 | 4 | 86 | 93 |
| 12 | 0 | 2 | 0 | 38 | 3 | 92 | 46 |
| 13 | 18 | 0 | 100 | 14 | 4 | 71 | 86 |
| 14 | 0 | 0 | 100 | 16 | 16 | 0 | 50 |
| 15 | 0 | 0 | 100 | 22 | 14 | 36 | 68 |
| 16 | 17 | 1 | 94 | 26 | 2 | 92 | 93 |
| 17 | 7 | 3 | 57 | 17 | 0 | 100 | 79 |
| 18 | 18 | 1 | 94 | 27 | 9 | 67 | 81 |
| 19 | 25 | 0 | 100 | 22 | 9 | 59 | 80 |
| 20 | 0 | 2 | 0 | 23 | 8 | 65 | 33 |
| 21 | 0 | 2 | 0 | 21 | 2 | 90 | 45 |
| 22 | 14 | 0 | 100 | 46 | 2 | 96 | 98 |
| 23 | 14 | 0 | 100 | 35 | 0 | 100 | 100 |
| 24 | 7 | 5 | 29 | 37 | 7 | 81 | 55 |
| 25 | 27 | 0 | 100 | 27 | 1 | 96 | 98 |
| 26 | 9 | 10 | -11 | 8 | 3 | 63 | 26 |
| 27 | 7 | 4 | 43 | 12 | 8 | 33 | 38 |
| 28 | 4 | 3 | 25 | 7 | 4 | 43 | 34 |
| 29 | 16 | 0 | 100 | 18 | 8 | 56 | 78 |
| 30 | 11 | 2 | 82 | 15 | 3 | 80 | 81 |
| 31 | 11 | 0 | 100 | 21 | 2 | 90 | 95 |
| 32 | 18 | 0 | 100 | 11 | 2 | 82 | 91 |
| 33 | 10 | 4 | 60 | 11 | 3 | 73 | 66 |
| 34 | 7 | 3 | 57 | 15 | 5 | 67 | 62 |
| 35 | 0 | 1 | 0 | 8 | 3 | 63 | 31 |
| 36 | 26 | 0 | 100 | 52 | 8 | 85 | 92 |
| 37 | 27 | 0 | 100 | 9 | 0 | 100 | 100 |
| 38 | 5 | 0 | 100 | 27 | 4 | 85 | 93 |
| 39 | 7 | 4 | 43 | 11 | 5 | 55 | 49 |
| 40 | 0 | 0 | 0 | 12 | 3 | 75 | 38 |
| 41 | 5 | 0 | 100 | 14 | 5 | 64 | 82 |
| 42 | 4 | 1 | 75 | 10 | 6 | 40 | 58 |
| 43 | 8 | 0 | 100 | 15 | 3 | 80 | 90 |
| 44 | 2 | 4 | -100 | 14 | 5 | 64 | -18 |
| 45 | 3 | 1 | 67 | 9 | 4 | 56 | 61 |
| 46 | 2 | 1 | 50 | 6 | 3 | 50 | 50 |
| 47 | 2 | 2 | 0 | 10 | 3 | 70 | 35 |
| 48 | 22 | 2 | 91 | 43 | 6 | 86 | 88 |
| 49 | 3 | 0 | 100 | 21 | 3 | 86 | 93 |
| 50 | 17 | 2 | 88 | 17 | 4 | 76 | 82 |
| 51 | 4 | 0 | 100 | 12 | 1 | 92 | 96 |
| 52 | 7 | 0 | 100 | 27 | 2 | 93 | 96 |
| 53 | 8 | 0 | 100 | 35 | 2 | 94 | 97 |
| 54 | 13 | 0 | 100 | 12 | 0 | 100 | 100 |
| 55 | 5 | 0 | 100 | 8 | 0 | 100 | 100 |
| 56 | 9 | 0 | 100 | 21 | 3 | 86 | 93 |
| 57 | 15 | 2 | 87 | 28 | 5 | 82 | 84 |
| 58 | 0 | 0 | 0 | 7 | 2 | 71 | 36 |
| 59 | 23 | 0 | 100 | 4 | 0 | 100 | 100 |
| 60 | 5 | 0 | 100 | 9 | 3 | 67 | 83 |

*: The overall reduction is calculated by averaging the reduction percentages of the 2D gaps and of the 2D step-offs.

Table A2: The results of the 3D measurements (the mean 3D step-off, 3D mean gap and the total gap area) and the percentage of overall reduction of 60 patients.

|  | **3D step-off** | | | **3D gap** | | | **Total gap area** | | | **Overall** |
| --- | --- | --- | --- | --- | --- | --- | --- | --- | --- | --- |
| **Patient** | **Pre-operative (in mm)*** | **Post-operative (in mm)*** | **Reduction (in %)** | **Pre-operative (in mm)*** | **Post-operative (in mm)*** | **Reduction (in %)** | **Pre-operative (in mm^2^)** | **Post-operative (in mm^2^)** | **Reduction (in %)** | **Reduction (in %) ***** |
| 1 | 3 (0 - 10) | 2 (0 - 5) | 43 | 25 (3 - 83) | 4 (1 - 6) | 85 | 1175 | 24 | 98 | 75 |
| 2 | 6 (0 - 15) | 3 (0 - 7) | 52 | 9 (3 - 20) | 7 (2 - 10) | 25 | 799 | 743 | 7 | 28 |
| 3 | 7 (0 - 25) | 3 (0 - 8) | 56 | 21 (8 - 33) | 7 (3 - 10) | 66 | 1102 | 367 | 67 | 63 |
| 4 | 7 (0 - 18) | 3 (0 - 9) | 61 | 26 (4 - 43) | 8 (3 - 13) | 67 | 636 | 178 | 72 | 67 |
| 5 | 8 (0 - 15) | 3 (0 - 7) | 66 | 11 (3 - 15) | 4 (0 - 8) | 65 | 380 | 128 | 66 | 66 |
| 6 | 5 (0 - 9) | 3 (0 - 9) | 35 | 9 (3 - 15) | 7 (4 - 10) | 23 | 541 | 401 | 26 | 28 |
| 7 | 6 (0 - 12) | 1 (0 - 2) | 86 | 9 (1 - 19) | 2 (1 - 3) | 77 | 738 | 82 | 89 | 84 |
| 8 | 7 (0 - 20) | 2 (0 - 13) | 70 | 12 (4 - 21) | 3 (0 - 12) | 78 | 1083 | 205 | 81 | 76 |
| 9 | 14 (0 - 34) | 5 (0 - 18) | 65 | 28 (13 - 39) | 10 (2 - 20) | 64 | 961 | 196 | 80 | 70 |
| 10 | 4 (0 - 14) | 1 (0 - 3) | 82 | 8 (1 - 17) | 1 (0 - 4) | 82 | 559 | 104 | 81 | 82 |
| 11 | 0 | 0 | N/A ** | 20 (2 - 45) | 4 (1 - 9) | 79 | 595 | 23 | 96 | 88 **** |
| 12 | 7 (0 - 31) | 1 (0 - 2) | 88 | 17 (3 - 41) | 2 (1 - 5) | 88 | 895 | 173 | 81 | 86 |
| 13 | 8 (0 - 15) | 1 (0 - 3) | 84 | 9 (3 - 19) | 2 (0 - 3) | 82 | 204 | 48 | 77 | 81 |
| 14 | 4 (0 - 12) | 3 (0 - 8) | 32 | 8 (2 - 17) | 5 (2 - 9) | 34 | 890 | 604 | 32 | 33 |
| 15 | 6 (0 - 19) | 3 (0 - 12) | 52 | 13 (4 - 19) | 7 (0 - 14) | 48 | 1029 | 745 | 28 | 43 |
| 16 | 7 (0 - 16) | 1 (0 - 4) | 81 | 11 (1 - 18) | 3 (0 - 6) | 73 | 982 | 223 | 77 | 77 |
| 17 | 8 (0 - 11) | 2 (0 - 3) | 78 | 7 (0 - 16) | 2 (0 - 3) | 79 | 350 | 0 | 100 | 86 |
| 18 | 9 (0 - 19) | 3 (0 - 9) | 64 | 14 (2 - 26) | 5 (0 - 11) | 64 | 1137 | 431 | 62 | 63 |
| 19 | 7 (0 - 30) | 2 (0 - 6) | 67 | 11 (1 - 33) | 4 (1 - 6) | 67 | 563 | 236 | 58 | 64 |
| 20 | 11 (0 - 26) | 2 (0 - 7) | 84 | 26 (8 - 34) | 6 (2 - 8) | 79 | 439 | 46 | 89 | 84 |
| 21 | 4 (0 - 11) | 2 (0 - 4) | 61 | 7 (2 - 11) | 4 (1 - 7) | 46 | 645 | 104 | 84 | 64 |
| 22 | 15 (0 - 40) | 3 (0 - 8) | 82 | 17 (4 - 47) | 3 (0 - 8) | 81 | 1505 | 75 | 95 | 86 |
| 23 | 19 (4 - 27) | 2 (0 - 3) | 91 | 25 (0 - 58) | 4 (1 - 9) | 84 | 1383 | 45 | 97 | 91 |
| 24 | 7 (0 - 30) | 2 (0 - 9) | 80 | 14 (1 - 33) | 3 (0 - 9) | 77 | 1130 | 376 | 67 | 75 |
| 25 | 9 (0 - 25) | 4 (0 - 12) | 55 | 16 (5 - 39) | 1 (0 - 1) | 96 | 797 | 0 | 100 | 84 |
| 26 | 10 (0 - 27) | 6 (0 - 25) | 43 | 13 (3 - 21) | 7 (2 - 24) | 47 | 1105 | 214 | 81 | 57 |
| 27 | 5 (0 - 16) | 3 (0 - 11) | 42 | 12 (4 - 16) | 8 (3 - 11) | 37 | 818 | 683 | 16 | 32 |
| 28 | 7 (0 - 18) | 3 (0 - 11) | 48 | 11 (3 - 21) | 6 (2 - 11) | 40 | 887 | 192 | 78 | 55 |
| 29 | 9 (0 - 27) | 5 (0 - 12) | 37 | 7 (1 - 29) | 7 (2 - 13) | 13 | 998 | 452 | 55 | 35 |
| 30 | 3 (0 - 18) | 2 (0 - 5) | 51 | 11 (1 - 55) | 3 (1 - 8) | 72 | 588 | 24 | 96 | 73 |
| 31 | 8 (0 - 20) | 3 (0 - 7) | 64 | 14 (1 - 24) | 4 (1 - 10) | 70 | 1375 | 136 | 90 | 75 |
| 32 | 4 (1 - 7) | 1 (0 - 2) | 77 | 7 (0 - 18) | 1 (0 - 2) | 82 | 40 | 0 | 100 | 86 |
| 33 | 3 (0 - 10) | 2 (0 - 4) | 32 | 9 (2 - 14) | 4 (1 - 6) | 58 | 927 | 434 | 53 | 48 |
| 34 | 5 (0 - 14) | 2 (0 - 7) | 51 | 15 (6 - 22) | 8 (2 - 12) | 49 | 587 | 285 | 52 | 51 |
| 35 | 2 (0 - 11) | 1 (0 - 7) | 43 | 2 (0 - 11) | 1 (0 - 7) | 43 | 492 | 312 | 37 | 41 |
| 36 | 12 (0 - 36) | 1 (0 - 2) | 95 | 32 (3 - 62) | 1 (1 - 2) | 96 | 1528 | 200 | 87 | 93 |
| 37 | 5 (0 - 10) | 2 (0 - 5) | 60 | 7 (3 - 13) | 1 (0 - 4) | 80 | 156 | 0 | 100 | 80 |
| 38 | 6 (0 - 13) | 3 (0 - 13) | 43 | 9 (1 - 17) | 5 (1 - 15) | 41 | 723 | 282 | 61 | 48 |
| 39 | 7 (0 - 20) | 1 (0 - 3) | 87 | 17 (4 - 41) | 3 (0 - 6) | 86 | 640 | 362 | 43 | 72 |
| 40 | 3 (0 - 6) | 2 (0 - 4) | 36 | 8 (4 - 13) | 5 (1 - 9) | 38 | 301 | 50 | 83 | 52 |
| 41 | 23 (0 - 66) | 2 (0 - 5) | 92 | 18 (5 - 52) | 3 (0 - 9) | 81 | 2188 | 48 | 98 | 90 |
| 42 | 3 (0 - 9) | 2 (0 - 6) | 22 | 6 (1 - 10) | 5 (1 - 7) | 21 | 337 | 192 | 43 | 29 |
| 43 | 8 (0 - 21) | 4 (0 - 8) | 56 | 13 (7 - 20) | 6 (2 - 10) | 54 | 865 | 162 | 81 | 64 |
| 44 | 3 (0 - 10) | 2 (0 - 4) | 46 | 6 (2 - 10) | 3 (1 - 4) | 54 | 368 | 106 | 71 | 57 |
| 45 | 3 (0 - 8) | 2 (0 - 7) | 31 | 9 (1 - 13) | 6 (0 - 10) | 28 | 332 | 213 | 36 | 32 |
| 46 | 3 (0 - 7) | 1 (0 - 4) | 49 | 6 (2 - 9) | 3 (1 - 5) | 52 | 226 | 79 | 65 | 55 |
| 47 | 4 (0 - 8) | 3 (0 - 7) | 35 | 5 (0 - 10) | 3 (0 - 9) | 45 | 455 | 292 | 36 | 39 |
| 48 | 9 (0 - 33) | 5 (0 - 11) | 47 | 15 (3 - 34) | 7 (3 - 12) | 54 | 1495 | 391 | 74 | 58 |
| 49 | 5 (0 - 12) | 2 (0 - 7) | 58 | 9 (2 - 14) | 5 (0 - 11) | 50 | 713 | 199 | 72 | 60 |
| 50 | 6 (0 - 16) | 2 (0 - 6) | 76 | 13 (5 - 21) | 6 (2 - 8) | 55 | 507 | 63 | 88 | 73 |
| 51 | 6 (0 - 16) | 2 (0 - 5) | 65 | 12 (5 - 20) | 5 (2 - 7) | 60 | 721 | 187 | 74 | 66 |
| 52 | 7 (1 - 24) | 1 (0 - 2) | 88 | 16 (2 - 41) | 1 (0 - 3) | 92 | 734 | 4 | 99 | 93 |
| 53 | 14 (0 - 31) | 2 (0 - 5) | 85 | 12 (2 - 29) | 2 (0 - 6) | 80 | 1083 | 29 | 97 | 87 |
| 54 | 6 (0 - 19) | 2 (0 - 3) | 74 | 13 (2 - 50) | 2 (0 - 3) | 84 | 636 | 65 | 90 | 83 |
| 55 | 8 (0 - 22) | 5 (0 - 18) | 36 | 18 (3 - 35) | 16 (7 - 20) | 9 | 1622 | 145 | 91 | 45 |
| 56 | 6 (1 - 12) | 2 (0 - 5) | 58 | 11 (6 - 16) | 3 (2 - 5) | 74 | 366 | 84 | 77 | 70 |
| 57 | 7 (1 - 15) | 4 (0 - 12) | 37 | 15 (6 - 20) | 10 (5 - 14) | 31 | 1031 | 196 | 81 | 50 |
| 58 | 2 (0 - 5) | 1 (0 - 3) | 56 | 4 (2 - 5) | 3 (1 - 3) | 34 | 297 | 133 | 55 | 48 |
| 59 | 8 (1 - 16) | 1 (0 - 1) | 89 | 21 (4 - 48) | 2 (1 - 3) | 92 | 369 | 0 | 100 | 94 |
| 60 | 4 (0 - 9) | 1 (0 - 3) | 59 | 6 (2 - 8) | 3 (1 - 4) | 52 | 255 | 155 | 39 | 50 |

*: Variables are denoted as the average value (range).

**: Percentage could not be calculated because this patient did not have a preoperative step-off, so it was not possible to assess the reduction in the step-off.

***: The overall reduction was calculated by averaging the percentages of the reductions in the 3D gaps, 3D step-offs and total gap areas.

****: The overall reduction was calculated by only using the reduction in the total gap area and the reduction in the gap.
