# Supplementary material for: Introduction of a three-dimensional computed tomography measurement method for acetabular fractures
Source: PLoS One. 2019 Jun 19;14(6):e0218612. doi: 10.1371/journal.pone.0218612 (PMC6583999; doi:10.1371/journal.pone.0218612)
Supplement: S1 Text — Validation of the gap and step-off measurements. (DOCX) [file pone.0218612.s003.docx]

# Supplementary materials III: Validation of gap and step-off measurements

To validate the measurement method, a phantom test was performed to test the accuracy of the measurements. A 3D printed pelvic model was used for this (Figure A2). The model was printed using selective laser sintering (SLS) and the model was printed from polyamide material. A CT scan (with a slice thickness of 0.6 mm) was made of the complete model and after that the model was sawn in two parts. For this a 1 mm thick saw blade was used and the printed 3D model was sawn in two pieces with the cuts through the acetabulum. The two parts were put together with a known step-off and a gap present and a new CT scan was made (with a slice thickness of 0.6 mm). The maximum fracture gaps and step-offs were measured on axial, coronal and sagittal CT slices, in consensus by 3 observers (2 trauma surgeons and 1 technical physician) (Figure A3). The intact 3D model was used as a template for the virtual reposition. After this, the 3D gap and step-off were calculated.

The actual gap was 15 mm and the step-off was 10 mm (Table A3). The 2D gap, as measured on axial, coronal and sagittal CT slices of this phantom, was 10.4 mm and the 2D step-off was 8.3 mm. In 3D the gap was 15.3 mm and the step-off was 10.8 mm. The phantom test showed a deviation in the 3D gap and 3D step-off within 1 mm from the actual values whereas the 2D gap and step-off measurements underestimated the actual values. For the 2D measurements the observer has to choose the slice and location where the gap and step-off are the largest. For the 3D measurements this is not necessary, the observer only has to identify the fracture lines. This can explain the difference between the 2D and 3D measurements. Additionally, the 2D measurements depend on the orientation of the CT slices with respect to the fracture lines and for the 3D measurements this has no influence on the values.


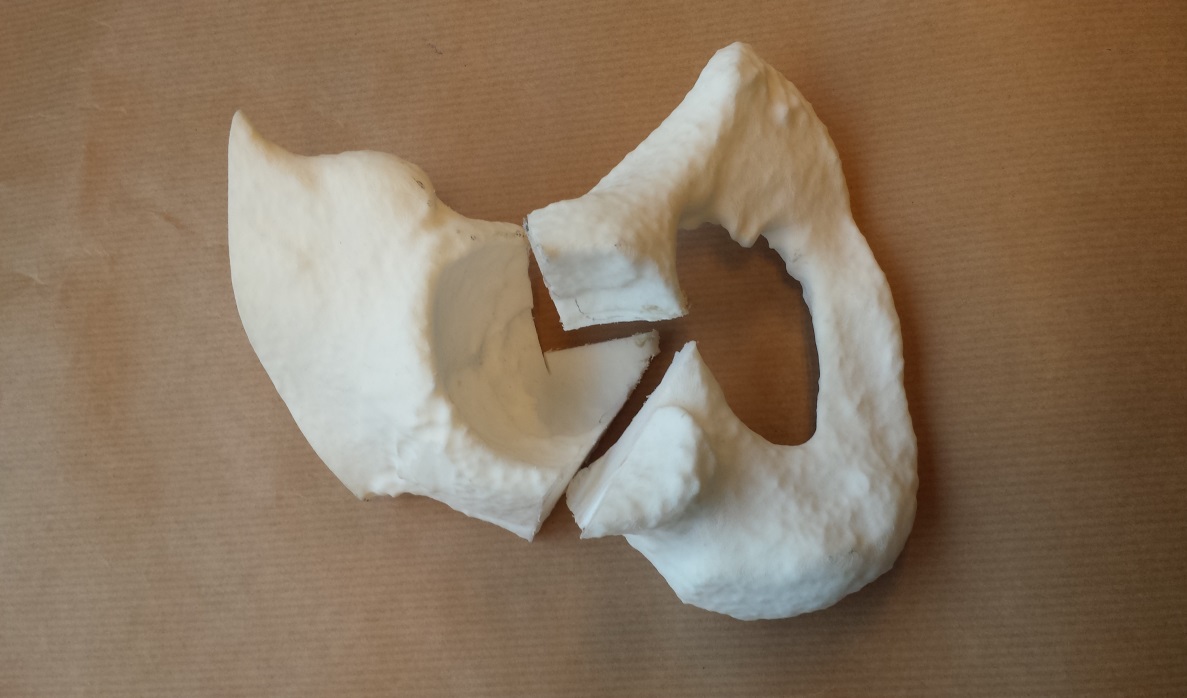

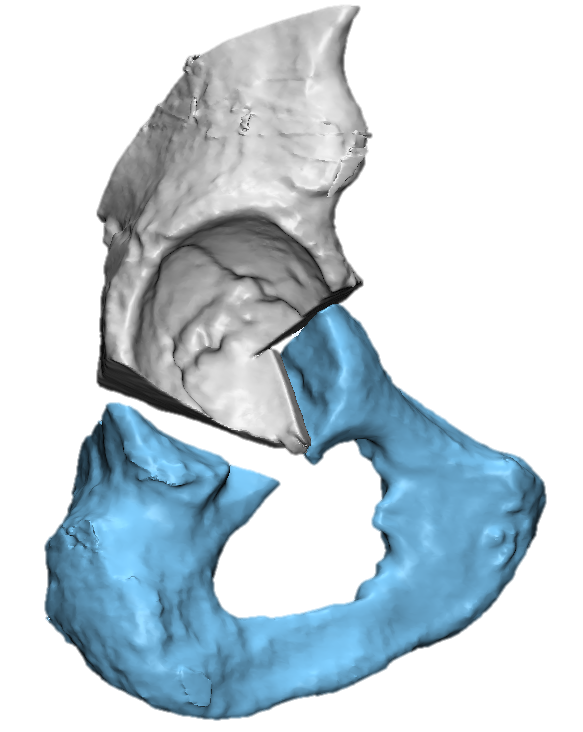


Figure A2: The phantom model, used to validate the 3D gap and 3D step-off measurement method. Left: The printed model, right: The virtual 3D model, based on the CT scan.


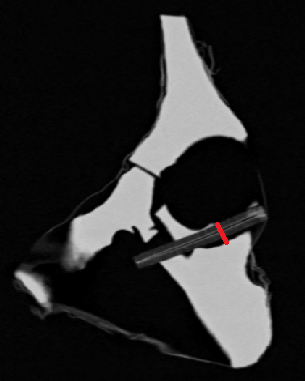

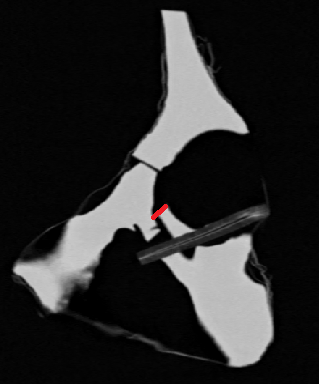

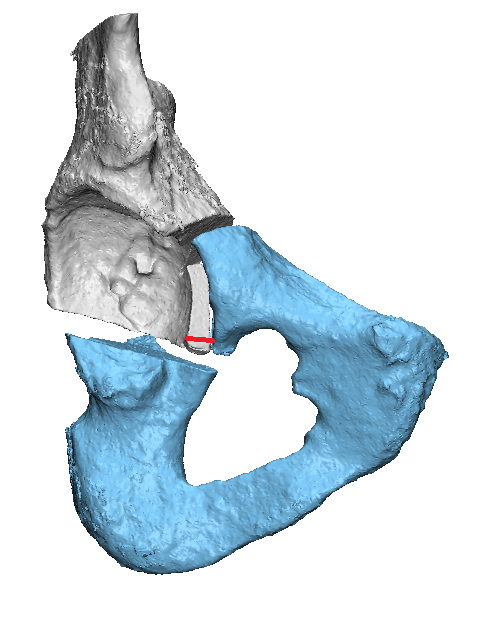

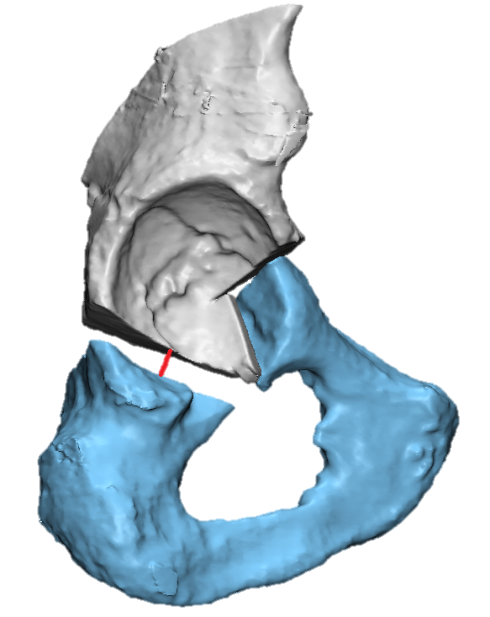


Figure 3A: The 2D gap (left) and step-off (right) measurements on the CT scan (top) and the 3D gap (left) and step-off (right) measurements on the 3D model (bottom).

Table A3: The results of the phantom test. The maximum values were used.

|  | **Actual measurements** | **2D measurements** | **3D measurements** |
| --- | --- | --- | --- |
| **Gap** | 15 mm | 10.4 mm | 15.3 mm |
| **Step-off** | 10 mm | 8.3 mm | 10.8 mm |
