# Supplementary material for: Introduction of a three-dimensional computed tomography measurement method for acetabular fractures
Source: PLoS One. 2019 Jun 19;14(6):e0218612. doi: 10.1371/journal.pone.0218612 (PMC6583999; doi:10.1371/journal.pone.0218612)
Supplement: S2 Text — A complete protocol for the 3D analysis, with all the measurements of a patient treated for an acetabulum fracture. (DOCX) [file pone.0218612.s004.docx]

# Supplementary materials IV: Postoperative evaluation of acetabular fractures

A postoperative evaluation of the acetabular fracture was performed for all patients, in order to determine the percentage of the reduction after surgery. A case example is shown below (Figure

A1).


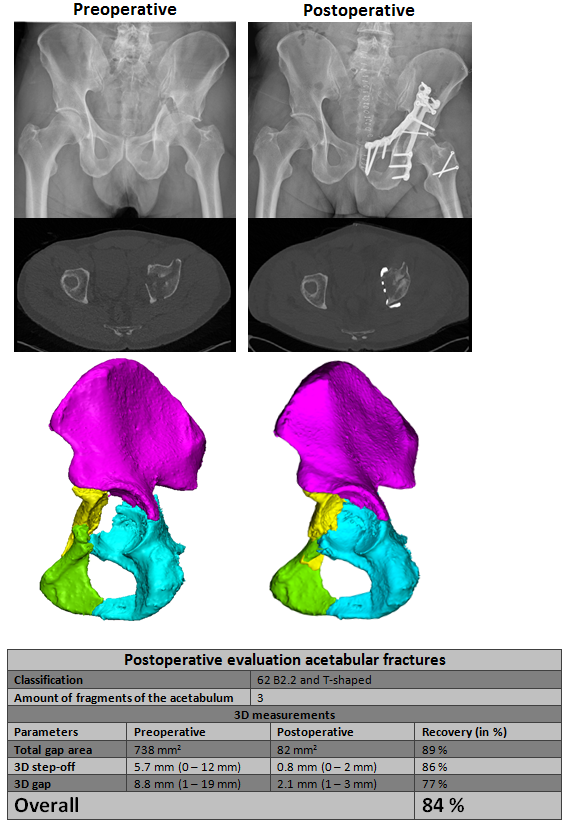


Figure A1: An example of the postoperative evaluation of an acetabular fracture. Left: The preoperative situation and right: The postoperative situation, with the plain radiographs (top), an axial CT slice at the dome (middle) and the pre- and postoperative 3D models (bottom).
